# Microcurrent therapy as the nonpharmacological new protocol against Alzheimer’s disease

**DOI:** 10.3389/fnagi.2024.1344072

**Published:** 2024-01-18

**Authors:** Eun Ho Kim, Won Seok Lee, Jae Hee Lee, Dong Rak Kwon

**Affiliations:** ^1^Department of Biochemistry, School of Medicine, Daegu Catholic University, Daegu, Republic of Korea; ^2^Department of Rehabilitation Medicine, School of Medicine, Daegu Catholic University, Daegu, Republic of Korea

**Keywords:** microcurrent therapy, Alzheimer’s disease, β-Amyloid, BACE1, MAPK signaling pathways

## Abstract

**Introduction:**

Alzheimer’s disease (AD) poses an increasing global health challenge and is marked by gradual cognitive deterioration, memory impairment, and neuroinflammation. Innovative therapeutic approaches as non-pharmacological protocol are urgently needed with side effect risk of drugs. Microcurrent therapy, a non-invasive modality involving low-level electrical currents, has emerged as a potential solution to address AD’s complex pathogenesis. This study investigates the optimal application of microcurrent therapy as a clinical protocol for AD, utilizing a comprehensive approach that integrates behavioral assessments and neuroinflammation evaluation in a mouse model of dementia.

**Methods and results:**

The results reveal that microcurrent therapy holds promise in ameliorating memory impairment and reducing neuroinflammation in AD. Behavioral assessments, including the Novel Object Recognition Test (NOR) and Radial Arm Maze Test (RAM), demonstrated improved cognitive function following microcurrent therapy. Furthermore, microcurrent therapy inhibited expression of neuroinflammatory proteins, including ionized calcium binding adaptor molecule 1 (Iba1), and glial fibrillary acidic protein (GFAP) in current-treated group. Mechanistic insights suggest that microcurrent therapy may modulate neuroinflammation through the regulation of MAPK signaling pathways.

**Conclusion:**

This study emphasizes the prospect of microcurrent therapy as a safe and efficacious non-pharmacological strategy for Alzheimer’s disease (AD), providing optimism to the countless individuals impacted by this debilitating ailment. These results contribute to the developments of an innovative clinical protocol for AD and recovery from neurological injury, underscoring the significance of investigating unconventional therapeutic approaches for addressing this complex condition.

## Highlights

- The goal of this manuscript was to find optimal treatment duration for microcurrent stimulation in AD mouse models.
- 3-, 6- and 12-h stimulation periods were used to verify efficacy of the treatment.

## Introduction

Alzheimer’s disease (AD), an increasingly prevalent and devastating neurodegenerative condition, poses a significant global health challenge (Knopman et al., 2021).

AD is a multifaceted neurological condition marked by gradual cognitive deterioration, memory issues, and neuroinflammation (Masters et al., 2015). Despite decades of research, there remains an unmet need for effective treatments that can modify the course of this disease. Among the emerging possibilities, current therapy has garnered attention for its potential in addressing the cognitive decline associated with AD (Kim et al., 2009, 2019; Sabel et al., 2021; Liu et al., 2022). During current therapy, adverse effects indeed range from itching, tingling and burning sensations to redness beneath the stimulated areas (Bikson et al., 2016; Russo et al., 2017). Microcurrent stimulation therapy is a treatment method that uses a current of less than 1,000 μA, which can hardly be felt by the human body, and is measured in mA units (Koh, 2018; Kim et al., 2021). Microcurrent therapy, which entails delivering low-level electrical currents to targeted areas of the brain, shows potential for alleviating the cognitive symptoms and neuroinflammation linked to AD (Sabel et al., 2021; Liu et al., 2022). By assessing the ideal duration and efficacy of microcurrent therapy in this study, this investigation will contribute with valuable insights Microcurrent stimulation commonly applied in wound dressing has several physiological benefits such as cell growth and migration, suppressing the inflammatory responses, angiogenesis, promotion of wound healing, and, in some cases, depression (Yu et al., 2014; Hunckler and de Mel, 2017). In addition, microcurrent stimulation may positively affect post-traumatic memory loss (Childs and Crismon, 1988; Yu et al., 2014; Yennurajalingam et al., 2018). A study reported a considerable increase in the synthesis of the bone-forming protein BMP-4 with microcurrent stimulation followed by tibia fracture in rabbits (Cho, 2010). The exploration of current therapy in AD is motivated by its demonstrated effectiveness in modulating neural activity, reducing oxidative stress, and promoting cellular regeneration (Wu et al., 2022). Additionally, current therapy has shown anti-inflammatory properties, which may prove beneficial in addressing the neuroinflammatory component of AD (Andrade et al., 2016; Thair et al., 2017).

One crucial aspect of AD involves the buildup of beta-amyloid plaques within the brain, which leads to impaired neuronal function and cognitive decline (Jagust, 2018). Although numerous contributing factors exist (Haass and Selkoe, 2007; Cervellati et al., 2016; De Strooper and Karran, 2016), the abnormal buildup of Ab is thought to play a role in the onset and advancement of AD. Aβ is generated through the successive cleavage of Amyloid precursor protein (APP) by β-secretases, including BACE1, and the γ-secretase complex. There are two primary species, Aβ40 and Aβ42, with Aβ42 being the predominant component of Aβ plaques in the brains of AD patients (Iwatsubo et al., 1995; Blennow et al., 2006; Nelson et al., 2009). Current therapy has the potential to influence these pathological processes by facilitating the clearance of beta-amyloid deposits (Andrade et al., 2016; Thair et al., 2017).

Another compelling aspect of current therapy is its non-invasiveness and minimal side effects compared to other treatment modalities such as drugs. Traditional pharmacological interventions often come with adverse effects, making them less desirable for long-term management of Alzheimer’s disease (Haley, 2022). Current therapy’s safety profile is particularly attractive for elderly patients who may be more vulnerable to medication-related side effects.

To understand the optimal duration of microcurrent therapy for Alzheimer’s disease, we employed a comprehensive approach that includes behavioral assessments and neuroinflammation evaluation. Behavioral assessments may involve a battery of evaluations to measure cognitive function, memory retention, and overall neurological well-being (Chaves et al., 2011). Additionally, neuroinflammation markers such as Transforming growth factor (TGF-beta) may assess the impact of microcurrent therapy on reducing neuroinflammatory processes in the brain (Rauf et al., 2022).

The rationale behind investigating the duration of microcurrent therapy lies in striking the right balance between maximizing therapeutic benefits and minimizing potential risks. Prolonged exposure to microcurrent stimulation may offer the opportunity for cognitive improvements and it may facilitate deeper and more lasting effects on neuroinflammation, a critical factor in the progression of AD.

Therefore, this research underscores the importance of exploring nonpharmacological therapeutic modalities for AD, a condition that continues to challenge the medical community. Microcurrent therapy represents a non-invasive and potentially effective approach to mitigate the evolution of ADBy conducting a comprehensive analysis of the optimal treatment duration and its impact on behavioral and neuroinflammatory parameters, this study aims to assess the most effective utilization of microcurrent therapy as a clinical, non-pharmacological protocol for AD and find optimal treatment duration for microcurrent stimulation in AD mouse models.

## Materials and methods

### Chemicals and antibodies

Here is the information regarding the chemicals and antibodies utilized in the procedure: Amyloid-β (Santa Cruz, Dallas, TX, United States, sc-53822, 1:200), BACE1 (Invitrogen, Waltham, MA, United States, PA5-19952, 10 μg/mL), APP (Invitrogen, 14–9,749-82, 2.5 μg/mL), Bace1 (Invitrogen, PA5-19952, 1:1000), Iba-1 (abcam Cambridge, CAM, UK, ab178846, 1:2000), GFAP (BD Biosciences, Dickinson, ND, United States, BD-556328, 5 μg/mL), β-actin (Santa Cruz, sc-8432, 1:1000), phospho-JNK (Santa Cruz, sc-6254, 1:1000), JNK (Cell signaling, cs-9258, 1:1000) phospho-ERK (Santa Cruz, sc-7383, 1:1000), ERK (Cell signaling, cs-9102, 1:1000) phospho-p38 (Cell Signaling, Danvers, MA, United States, csD3F9, 1:1000), p38 (Santa Cruz, sc-535, 1:1000) Anti-goat mouse (Enzo Life Sciences, Farmingdale, NY, United States, ADI-SAB-100-J, 1:2000), Anti-goat rabbit (Enzo Life Sciences, ADI-SAB-300-J, 1:2000).

### Microcurrent therapy

To investigate the therapeutic effect of microcurrent, it was applied for the indicated times according to the specific period set for each group. Using connection wire from the microcurrent generator (Ecure, Busan, Korea), the current was delivered through a copper plate of the same size as the cage floor. This setup allowed the mice to receive the current through their feet touched to the floor, enabling the current to reach the brain. Since microcurrent is usually applied during active time of humans which is day time, the microcurrent was applied during the mice’s active time, which is the nighttime cycle for mice because of nocturnal animals. There are various waveforms of microcurrent (Kim et al., 2019), but revealed that all waveforms had an effect, but especially the step form waveform showed significant effects on both clinical parameters like cognition and proteins production related to Alzheimer’s disease in mice model. Therefore, we selected the microcurrent with the step form waveform (0, 1.5, 3, 5 V) with wave superposition. The intensity of the microcurrent was set to 1 μ A (250 ohm), the voltage was set to 5 V, and the basic frequency was set to 7 Hz with an additional 44KHZ frequency superimposition.

### Animals preparation

Approval for this study was obtained from the Institutional Animal Care and Use Committee (IACUC) of the Catholic University of Daegu School of Medicine (IRB no.: DCIAFCR-230329-06-YR). C57BL/6 female mice were purchased from JaBio (Daegu, Gyeongsangbuk-do, Korea) and the 5Xfad[B6STL-Tg(APPwFlLon,PSEN1*M146L*L286V)6799Vas/Mmjax] transgenic mice were purchased from the Jackson Laboratory (Bar Harbor, ME, United States). Female wild-type (WT) and Aβ-injected mice were employed and distributed into different groups. Microcurrent exposure commenced when the mice were 1.5 months old, at a stage when their brains had not yet fully matured. This timing was chosen because the genetic development of Intraneuronal Aβ aggregation began in Aβ-injected mice from this point onward. A bilateral intracerebroventricular (ICV) stereotaxic procedure was conducted, with injection coordinates established in reference to the bregma (coordinates: −1.0 ± 0.06 mm posterior to bregma, 1.8 ± 0.1 mm lateral to the sagittal suture, 2.4 mm in depth). This involved injecting 3 μL of 100 μM aggregated Aβ1–42 at a rate of 1 μL/min (Kim et al., 2016). The experiments were conducted in accordance with the approval obtained from the Institutional Animal Care and Use Committee (DCIAFCR-230329-06-YR). All relevant institutional and international guidelines for animal care were strictly followed. In the normal group, instead of injecting Aβ through the same procedure, the same amount of physiological saline is injected. After a one-week adaptation period, the mice were randomly assigned to either the i.c.v.-injected Aβ treated model group or the microcurrent + i.c.v.-injected Aβ treated model group (with 5 mice in each group). Additionally, mice were randomly assigned to either the normal control (NC) group or the microcurrent + NC group (again, with 5 mice in each group). Microcurrents were treated daily for one month for 3,6, and 12 h for each WT(saline+), Aβ injected group. 5xFAD, WT group was only microcurrent treated for 6 h daily for one month. After one month of timed microcurrent treatment, behavioral testing was conducted for 14 days before the mice were sacrificed for brain sampling. The mouse is 3 months old at the time of sacrifice.

#### β-amyloid administration

Aβ1-42 (Sigma-Aldrich; Merck KGaA, Darmstadt, Germany) is placed in 0.9% sterile saline and allowed it to dissolve, to a final concentration of 100 μM (Cetin et al., 2013; Kim et al., 2016). Control experiment is also performed injecting the same amount of albumin exogenous protein (100 μM, Sigma-Aldrich, Merck KGaA, Darmstadt, Germany) to verify the translational validity (Supplementary Figure S1). To promote fibril aggregation, the solution is incubated at 37°C with shaking for 7 days. First, anesthesia was induced by intraperitoneal injection of ketamine in the amount of 100 mg/kg. Make an incision in the skin of the mouse’s head to expose the bregma. Precisely at the coordinates, 3 μL of aggregated 100 μM Aβ1-42 and sterile saline was injected using a Hamilton syringe at a rate of 1 μL/min, the test group3 and sham-operated control group, respectively. Allow the mouse a week to recover after surgery.

### Novel object recognition test

Prior to and during the night preceding the training sessions, the mice were placed in a testing chamber with a twelve-hour light–dark cycle, kept at a constant temperature of 23 ± 1°C, and maintained at a humidity level ranging between 50–60%. They were provided with unrestricted access to both food and water.

Throughout the training phase, two circular filter units were situated within the chamber. Each unit had a height and diameter of 27 mm and 33 mm, respectively. The mice were allotted a period of ten minutes to investigate these objects. Subsequently, after one day, one of the objects was substituted with a plastic cone measuring 30 mm in height and 25 mm in diameter. Object recognition was defined based on the time the mice spent touching or sniffing the new object within a five-minute timeframe (Antunes and Biala, 2012). The training sessions and trials were documented and assessed employing EthoVision XT8.5 (Broadbent et al., 2010).

### Radial arm maze test

A neurocognitive Radial Arm Maze (RAM) assessment was conducted both prior to and following the administration of microcurrent treatment to diverse groups of mice. These groups encompassed untreated mice and non-transgenic (non-Tg) wild-type mice.

The spatial working memory of the mice was assessed using an eight-arm radial maze, as detailed in a prior study (Clark et al., 2015). A reward cup was positioned on a platform at the far end of each arm of the maze. The mice underwent initial 10-min habituation training sessions on the radial arm maze for three consecutive days to become acquainted with the maze.

Following this familiarization period, the mice were permitted to explore the open arms of the maze. Two arms were equipped with food rewards and were positioned.

135° apart from each other. The trial concluded once a mouse located and consumed the food reward from these baited arms. The recorded data included the number of arms visited by the mouse before finding the two baited arms, which also accounted for any revisits. A correct choice was registered only when the mouse approached and consumed from a baited food cup. Any instance of re-entering an arm that was either unbaited or had been previously baited was documented as a visiting error, indicating a lapse in spatial working memory.

The performance metrics were evaluated both prior to and following microcurrent treatment, and an analysis of variance (ANOVA) was conducted to assess them.

### Tissue preparation

Mice were sacrificed to obtain brain tissue for analysis following two months of microcurrent treatment after conducting behavioral tests. For histological analysis, the left hemispheres of three mice in each group were immersed in a 4% paraformaldehyde solution, while the right hemispheres were stored at −80°C. Subsequently, brain regions, specifically the hippocampus and entorhinal cortex, were dissected from three mice in each group, following the Paxinos and Franklin (Stone, 2012) atlas, for Western blotting analysis.

### Immunohistochemistry

Immunostaining of the sections was conducted using a Vectastain Elite ABC kit obtained from Vector Laboratories Inc., a U.S.-based company. To retrieve the antigen, the initial step involved immersing these sections in citrate buffer and then boiling them in water for half an hour. For immunoperoxidase labeling, a solution of 0.3% H_2_O_2_ in methanol was employed to block endogenous peroxidase activity, and this was carried out at room temperature for approximately one hour. In the context of immunohistochemistry, sections were first blocked with horse serum and subsequently incubated overnight at 4°C with either an anti-mouse BACE1, GFAP, Iba-1, or human Aβ antibody. Following this, sections were incubated with either mouse IgG or biotinylated goat anti-human antibodies at room temperature for 30 min. Subsequently, an immunoreaction with a peroxidase complex (avidin-biotin-based) was conducted at room temperature for half an hour. The DAB kit was employed to visualize the peroxidase reaction. Notably, for some sections in all experiments, the primary antibody was omitted, and these sections were counterstained using Harris hematoxylin before mounting.

### Analysis of western blot

Mouse brain tissues were dissected on ice and homogenized in RIPA lysis buffer (Biosesang, #R2002) before being supplemented with protease inhibitors (1 mM PMSF, 1 μg/mL aprotinin, 1 μg/mL leupeptin, and 1 mM Na_3_VO_4_) and then protein quantified using the Bradford assay. Protein samples (30 μg) were separated by SDS/polyacrylamide gel electrophoresis and transferred to a nitrocellulose membrane used as described previously.

The detection of antigen–antibody reactions was carried out using a chemidoc (Davinch-K, Seoul, Republic of Korea, #CAS-400SM) with an ECL kit (ThermoFisher, Waltham, MS, United States, #34095) on the membrane. The same conditions were applied to both WT and AD group blots during the detection process for each antibody.

### Statistical analysis

Statistical significance was assessed through one-way **ANOVA**, and **Tukey’s** method was applied to determine differences. Statistically significant distinctions were established when the *p*-value fell below 0.001 or 0.05 (*p < 0.05; **p < 0.01; ***p < 0.001).

## Results

### Microcurrent exposure improves memory function

**Figure 1:**
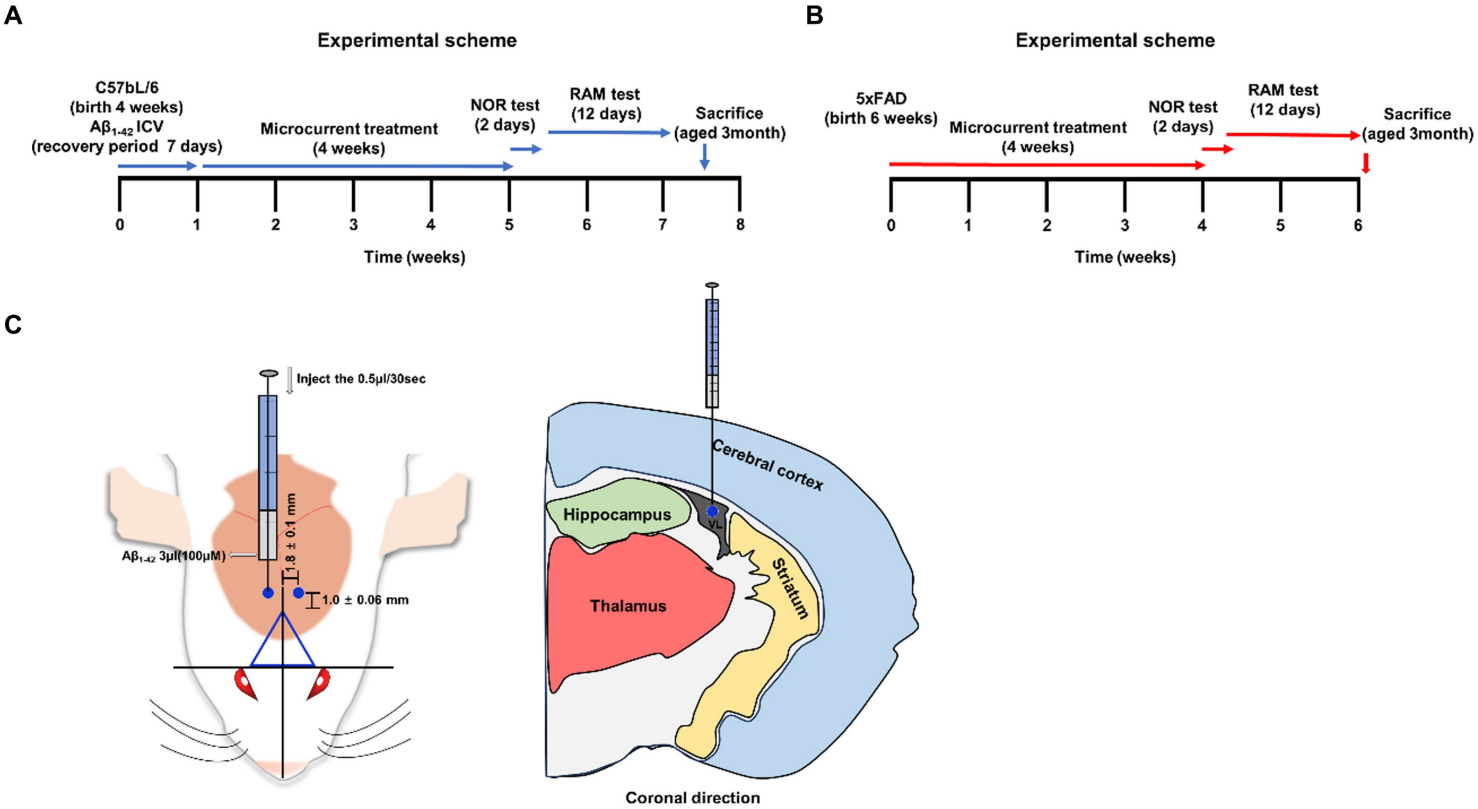
A schematic summar. **(A,B)** An illustrative representation of the experimental process. **(C)** The injection site, marked by a blue circle on the mouse brain (−1.0 ± 0.06 mm from bregma and 1.8 ± 0.1 mm sagittally). Visualization of brain sections in a coronal orientation depicting the bregma’s location.

**Figure 2:**
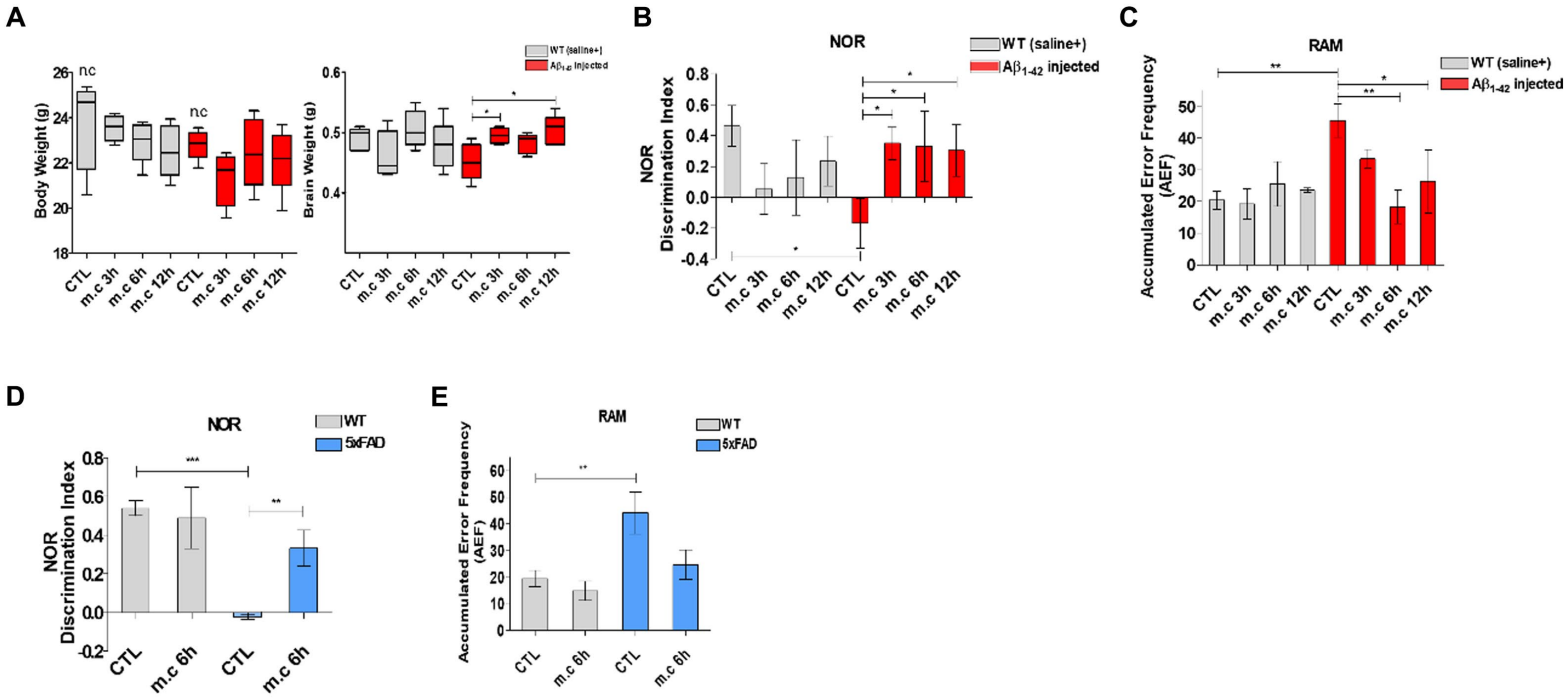
Microcurrent therapy attenuating memory impairment. **(A)** The body and brain tissues of the mice were weighed at the conclusion of the final experiment (after 8 weeks). **(B)** The novel object recognition task was performed on Aβ-injected mice and their control group (non-transgenic mice). The discrimination index was calculated as the percentage ratio of *TB/ (TA + TB)* × 100, where *TA* represents the familiar object and *TB* represents the novel object. **(C)** Spatial memory was assessed using the Radial Arm Maze test. **(D)** The novel object recognition task was perfomed on 5xFAD mice. **(E)** Spatial memory was assessed using the Radial Arm Maze test on 5xFAD mice. “Change” represents the percentage of non-overlapping frequency entries in comparison to the total entries within three arms. *p < 0.05, **p < 0.01, ***p < 0.001.

In this phase of the study, the objective was to explore the influence of microcurrent exposure on memory function in mice with Alzheimer’s disease (AD). An overview of the research methodology is provided in Figures 1A–C (Cetin et al., 2013; Kim et al., 2019). To evaluate the correlation between microcurrent treatment and cognitive improvement, mice were analyzed: one consisting of i.c.v.-injected Aβ treated mice (aged 3 months) and another of control mice (aged 3 months), 5xFAD transgenic mice (aged 3 months), wild-type mice (aged 3 months) each with and without microcurrent treatment. Similar body weight gain was observed during the whole life span in WT mice subjected to microcurrent treatment when compared with control WT mice (Figure 2A) (Fujiya et al., 2015). These groups underwent two spatial memory tests, specifically the RAM for the assessment of both short and long-term memory and NOR tests for long-term recognition memory, at specified time intervals of microcurrent exposure, and their results were compared (Figures 2B–E).

Following microcurrent treatment, the NOR showed the decrease slightly dependent the treatment period. And the RAM revealed a significant reduction in error frequency after 6 h and 12 h of microcurrent treatment, particularly in the i.c.v.-injected Aβ treated AD mice. This observation suggests that microcurrent exposure substantially ameliorated spatial memory impairment in AD mice while providing no discernible benefit to wild-type animals.

### Microcurrent therapy reduces brain Aβ deposition and protein expression

**Figure 3:**
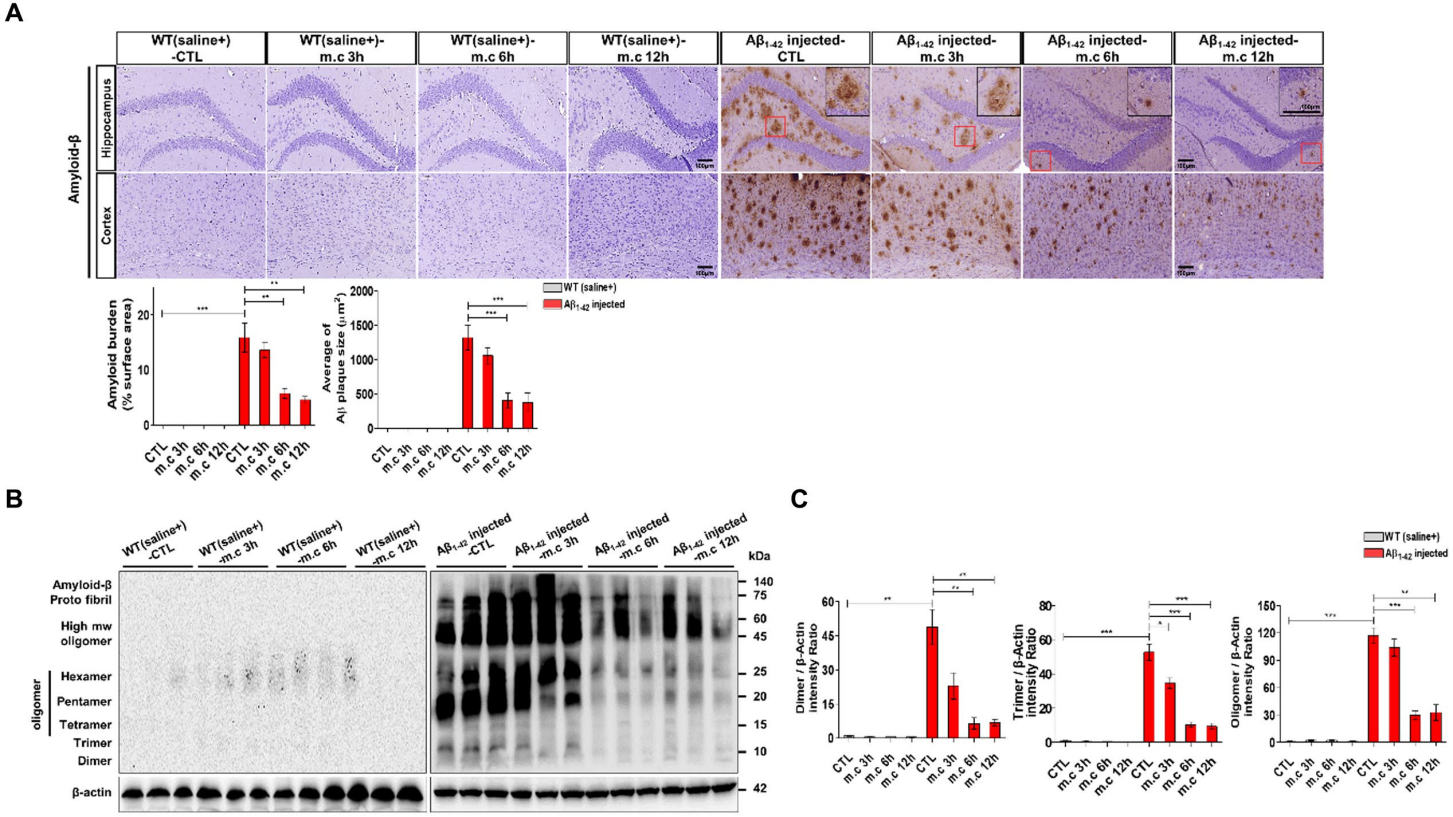
Microcurrent exposure lowers the deposition of Aβ in Aβ_1-42_-injected mice brain **(A)** Representative images depict Aβ deposition in the CA1 region of the hippocampus and entorhinal cortex in Aβ1-42-injected and WT(saline+) mice. *p < 0.05, **p < 0.01, ***p < 0.001 **(B)** Whole Brain tissue lysates (30 μg) from each *in vivo* treatment group were subjected to immunoblotting (IB) using the specified antibodies. **(C)** Western blot analysis was conducted to validate the expression differences in proteins.

Since Aβ accumulation in the cortex is one of the pathological characteristics of AD (Neuron. 6:487–498. 1991), the immunohistochemistry analysis of brain sections derived from Aβ-injected mice and wild-type (WT) mice following microcurrent exposure was used to evaluate the effect of MC administration on Aβ accumulation. In the i.c.v.-injected Aβ treated mice, there was a noticeable presence of Aβ deposits across their brains, whereas WT mice did not exhibit such deposition. Importantly, i.c.v.-injected Aβ treated mice with AD, who were exposed to microcurrent, showed a significant decrease in both the quantity and size of Aβ plaques at the 6-h and 12-h post-treatment time points (Figure 3A). Western blot analysis confirmed a decrease in Aβ levels following microcurrent therapy, consistent with the immunohistochemistry findings (Figures 3B,C) (Watt et al., 2013; Ghate et al., 2014).

### Effects of microcurrent on APP and BACE1 expression in the brain

**Figure 4:**
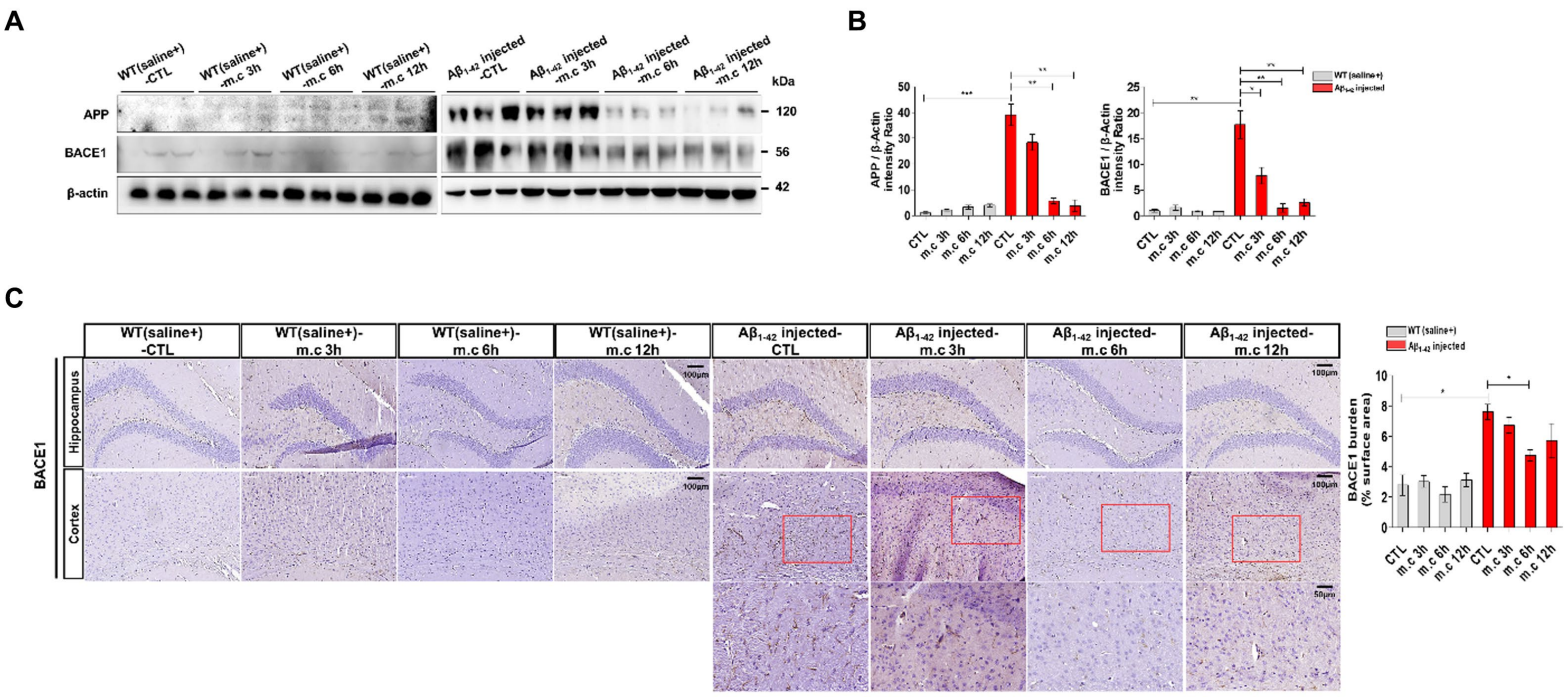
Impact of Microcurrent on differential expression of APP and BACE1 in the brains of WT(saline+) and Aβ_1-42_-injected mice. **(A)** Western blot analysis of APP conducted using whole brain tissues obtained from WT(saline+) and Aβ_1-42_-injected mice, with and without exposure to microcurrent. **(B)** Quantification of the expression differences in proteins through western blot analysis. **(C)** Representative images illustrating BACE1 levels in the CA1 region of the hippocampus and entorhinal cortex in Aβ_1-42_-injected and WT(saline+) mice. *p < 0.05, **p < 0.01, ***p < 0.001.

It was reported that the trafficking of APP and its processing enzymes, especially BACE1, is essential for APP processing and Aβ production (Haeffner-Cavaillon et al., 2015). We studied that western blotting was conducted on brain tissue to evaluate the impact of microcurrent exposure on APP processing and BACE1 (Harada et al., 2006; Nishitomi et al., 2006; Hebert et al., 2008; Kim et al., 2018). No significant differences were evident in the levels of APP in the entorhinal cortex and hippocampus of wild-type mice. Conversely, i.c.v.-injected Aβ treated mice displayed heightened levels of APP in their brains. Nevertheless, subsequent to microcurrent exposure, Aβ-injected mice demonstrated a decline in APP levels (Figures 4A,B). Immunostaining for BACE1 conducted on brain sections showed elevated BACE1 expression in i.c.v.-injected Aβ treated mice, notably within the entorhinal cortex and hippocampus, in contrast to WT mice (Figure 4C). However, i.c.v.-injected Aβ treated mice exposed to microcurrent displayed a noteworthy reduction in BACE1 expression across brain sections when compared to the control i.c.v.-injected Aβ treated mice with AD.

### Microcurrent exposure reduces neuroinflammation

**Figure 5:**
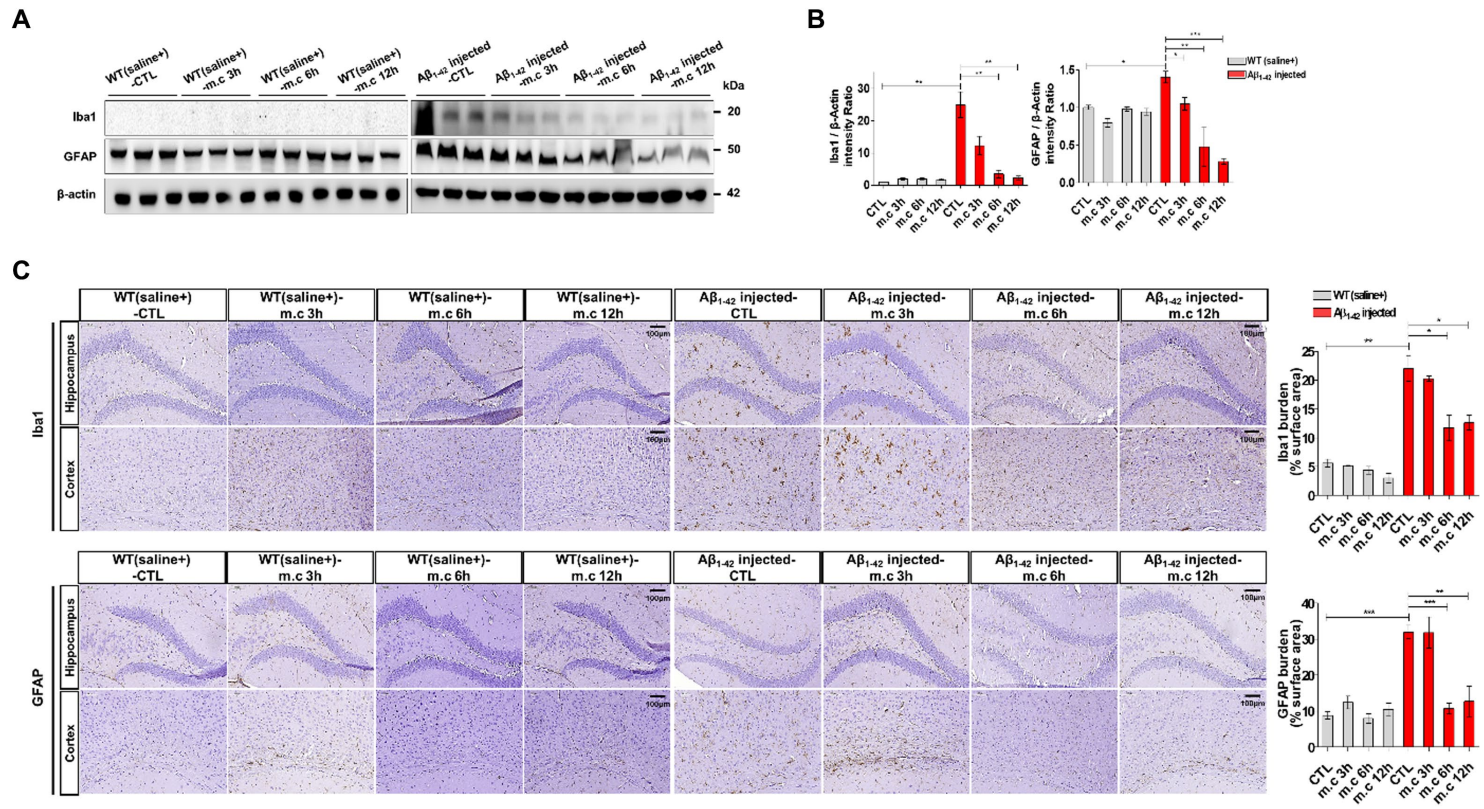
Reduced reactive astrocytes and active microglia. **(A)** Western blot analysis of GFAP and Iba1 in both cortex extracts and hippocampus samples from Aβ_1-42_-injected mice following microcurrent exposure, with actin serving as the loading control. **(B)** Quantification of the expression differences in proteins through western blot analysis. **(C)** Immunohistochemical staining of GFAP and Iba1 within the hippocampus and entorhinal cortex brain sections of Aβ1-42-injected and WT(saline+) mice after exposure to microcurrent. Immunoblotting and immunostaining quantifications were performed. *p < 0.05, **p < 0.01, ***p < 0.001.

To further investigate the neuroinflammation after MC treatment, we first used Western blotting to determine the protein levels of astrogliosis marker glial fibrillary acidic protein (GFAP) and microgliosis marker ionized calcium-binding adapter molecule 1 (IBA1) (Amalia, 2021; Muzio et al., 2021; Zhang et al., 2021). Increased GFAP expression, indicative of heightened astrocyte activation, was evident in i.c.v.-injected Aβ treated AD mice when compared to WT mice. Strongly positive GFAP signals were also observed in i.c.v.-injected Aβ treated AD mice. This elevated GFAP expression was further confirmed through Western blotting of brain homogenates from different brain regions. Notably, brain sections exposed to microcurrent treatment displayed a substantial reduction in GFAP expression, with the degree of reduction varying depending on the duration of treatment, as shown in Figures 5A,B. And the expression level was the lowest at the 6 h and 12 h after treatment. Similarly, immunoblotting data for Iba1, a marker for microglial activation, demonstrated lower expression in i.c.v.-injected Aβ treated mice exposed to microcurrent compared to untreated Aβ-injected mice (Figure 5C).

### Mechanisms of microcurrent therapy in AD-related phenotypes

**Figure 6:**
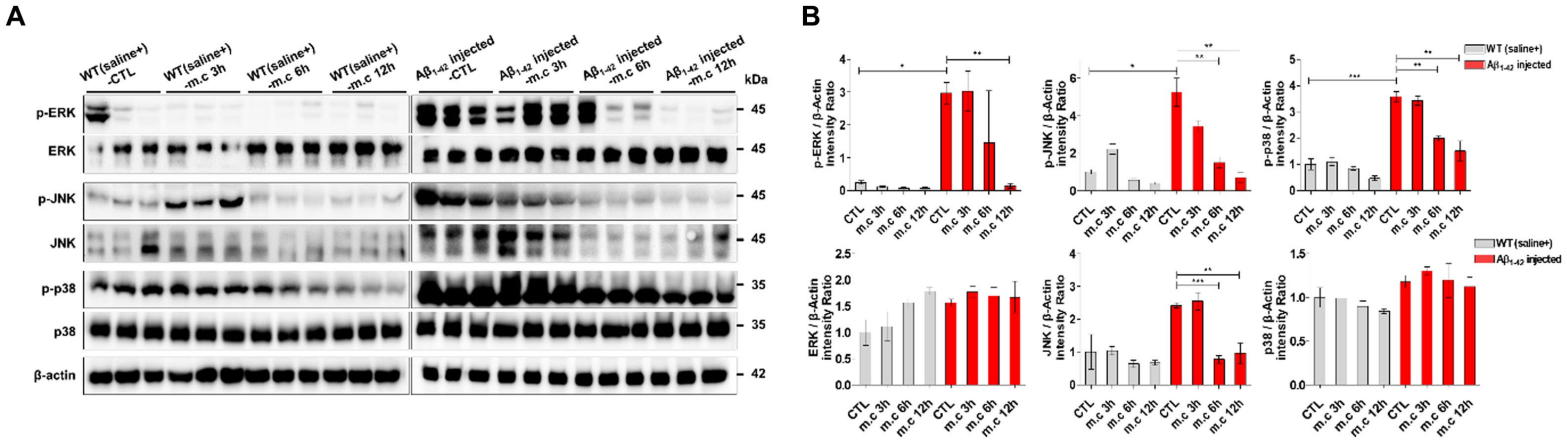
Inhibitory effects of Microcurrent on the phosphorylation of c- JNK, ERK, and p38. **(A)** Western blot analysis was employed to assess the expression levels of p-JNK, JNK, p-ERK, ERK, p-p38 and p38. **(B)** Verification of the differential expression of proteins was confirmed through western blot analysis. Immunoblotting quantifications were performed on all group. *p < 0.05, **p < 0.01, ***p < 0.001.

Given the critical role of mitogen-activated protein kinase (MAPK) pathways in regulating cellular processes that are affected in AD, the importance of MAPKs in disease pathogenesis is being increasingly recognized (Savage et al., 2002; Otth et al., 2003; Peel et al., 2004). All MAPK pathways are activated in vulnerable neurons in patients with AD suggesting that MAPK pathways are involved in the pathophysiology and pathogenesis of AD (Wang et al., 2014; Lee and Kim, 2017). As MAPK is considered to serve a key role in AD pathophysiology (Munoz and Ammit, 2010), our study investigated whether MC treatment affects MAPK signaling. Our results showed Western blot analysis revealed a significant reduction in the levels of phosphorylated JNK, phosphorylated ERK, and phosphorylated p38 following microcurrent treatment when compared to the control group (WT-control, Aβ_1-42_-injected control) (Figures 6A,B). These results indicate that microcurrent therapy could potentially exert its effects by influencing the modulation of the JNK/ERK/p38 MAPK signaling pathways, as shown in Figures 6A,B.

## Discussion

### Therapeutic potential of microcurrent therapy

Microcurrent therapy has garnered recognition for its capacity to regulate neural activity, mitigate oxidative stress, inducecellular regeneration, and demonstrate anti-inflammatory characteristics (Andrade et al., 2016; Thair et al., 2017). These mechanisms position it as a promising contender for treating AD.

A defining characteristic of AD is the buildup of beta-amyloid plaques, which play a role in neuronal dysfunction and the decline of cognitive function (Jagust, 2018; Knopman et al., 2021). Furthermore, the control of amyloid precursor protein (APP) processing is a significant factor in the production of Aβ in the brain. It has been established that the phosphorylation of APP and essential enzymes implicated in the proteolytic cleavage of APP is crucial in regulating the generation of Aβ (O’Brien and Wong, 2011; Zhang et al., 2011). This regulation can occur by modifying either the subcellular distribution of APP or the enzymatic functions of the secretases responsible for APP processing (Zhang et al., 2019). In accordance with the references, our data showed promise in enhancing the clearance of beta-amyloid deposits and the level of APP.

The non-invasive nature and minimal side effects of microcurrent therapy may make it particularly appealing for elderly patients who may be more vulnerable to medication-related adverse effects (Haley, 2022). This aspect of microcurrent therapy aligns with the need for safe and well-tolerated long-term treatment options for AD.

### Optimal duration of microcurrent therapy

Determining the optimal duration of microcurrent therapy is essential to decide the right balance between maximizing therapeutic benefits and minimizing potential risks (Bikson et al., 2016; Russo et al., 2017; Koh, 2018). Prolonged exposure to microcurrent stimulation may offer the opportunity for more substantial cognitive improvements and comprehensive neural regeneration. It may facilitate more profound and lasting effects on neuroinflammation, a critical factor in AD progression. Thus, we need to study the safety of extended-term impacts in clinical trial.

### Behavioral, neuroinflammatory assessment and amyloid-degrading enzymes (ADE)

This study showed a comprehensive approach, combining behavioral assessments and neuroinflammation evaluation, to understand the impact of microcurrent therapy on AD. Behavioral assessments, including the NOR and RAM, provided valuable insights into the cognitive improvements achieved through microcurrent therapy. NOR results showed a little reduction according to the treatment time points, but the RAM test demonstrated a reduction in error frequency at 6 h and 12 h of microcurrent exposure, suggesting enhanced spatial memory. These results emphasize the promise of microcurrent therapy in alleviating the cognitive impairments linked to AD.

Reduced expression of GFAP and Iba1, markers for astrocyte and microglial activation, respectively, in Aβ-injected AD mice exposed to microcurrent, suggests a potential anti-inflammatory effect of this therapy. These findings align with prior research that has underscored microcurrent therapy’s capacity to regulate neuroinflammation (Kim et al., 2021). Amyloid-degrading enzymes (ADE) was reported as its role in removing harmful amyloid moieties. In AD patients, the survival of the synapse and neuronal cell is directly influenced by insulin resistance and indirectly by insulin-degrading enzyme (IDE), which is likely a key player in Aβ catabolism (Nalivaeva et al., 2012). In addition, neprilysin (NEP) inhibits the progression of AD by degrading Aβ plaques (Jha et al., 2015). Amyloid fibrils in AD are formed from Aβ peptide, which results in isoforms of different length. The residue peptide Aβ (1–40) and (1–42) constitute the most abundant Aβ isoform in the brain and AD, respectively (Yu et al., 2021). Aβ (1–42) peptide had the role in AD in the central nervous system via more neurotoxic aggregated form (Wang et al., 2016; Zhang et al., 2016; Kasza et al., 2017; Mirza et al., 2021). Thus, targeting the inhibition of aggregation is one of most primary research aims in the AD field. So, we need to study MC might upregulate IDE and NEP and indirectly suppress the toxicity of Aβ (1–42) peptide.

### Mechanistic insights

The study also focused on the potential mechanisms underlying the therapeutic effects of microcurrent therapy. Additionally, the study revealed that the ERK/p38 MAPK pathway has the ability to control decreased the amount of Aβ deposits, and reduced spatial learning memory loss (Webber et al., 2005; Sarina et al., 2013). In a model of Alzheimer’s disease, it has been demonstrated that the cleavage and degradation of APP are regulated by JNK (Sarina et al., 2013). The recent research studies reported the therapeutic role of p38 MAPK in treating Alzheimer’s disease (AD) (Munoz and Ammit, 2010). And Stress-responsive MAP kinase pathways were activated in the brain of the Tg2576/PS1(P264L) AD model, and this activation was in accord to the age-dependent increase in amyloid deposition, tau phosphorylation, and loss of synaptophysin (Savage et al., 2002). The role of p38 and ERK in the cellular stress and mitotic signaling, respectively signifies their role in pathogenesis of AD, as evident in mitotic and oxidative insults (Webber et al., 2005). Our results of analysis of MAPK signaling pathways uncovered a reduction in the levels of phosphorylated JNK, ERK, and p38 MAPK subsequent to microcurrent therapy in accordance with the references. This indicates that microcurrent therapy could potentially achieve its effects by regulating MAPK signaling pathways, which play a role in both neuroinflammation and neurodegenerative mechanisms.

### Limitations and future directions

Although the results obtained from our study hold promise, it’s important to note that the study primarily centered around an AD mice model. Further research is needed to determine the applicability of these findings to human patients. Additionally, the optimal duration of microcurrent therapy needs to be validated in clinical settings. Subsequent studies need to investigate the extended-term impacts of microcurrent therapy, potential dose–response associations, and assess its safety profile in clinical trials involving individuals with AD. Moreover, understanding the precise mechanisms by which microcurrent therapy modulates neuroinflammation and cognitive function will provide deeper insights into its therapeutic potential.

In summary, this investigation provides valuable insights into the potential of microcurrent therapy as a non-invasive and promising non-pharmacological strategy for addressing cognitive decline and neuroinflammation in AD. These findings highlight the significance of exploring alternative therapeutic methods to address AD. Although additional research is required to validate these outcomes in clinical settings, microcurrent therapy shows potential as a valuable addition to the repertoire of treatments for AD.

## Data availability statement

The original contributions presented in the study are included in the article/Supplementary material, further inquiries can be directed to the corresponding author.

## Ethics statement

The animal study was approved by Approval for this study was obtained from the Institutional Animal Care and Use Committee (IACUC) of the Catholic University of Daegu School of Medicine (IRB no.: DCIAFCR-230329-06-YR). The study was conducted in accordance with the local legislation and institutional requirements.

## Author contributions

EK: Data curation, Formal analysis, Investigation, Methodology, Project administration, Resources, Software, Supervision, Validation, Visualization, Writing – original draft, Writing – review & editing. WL: Data curation, Formal analysis, Investigation, Methodology, Resources, Validation, Visualization, Writing – original draft, Writing – review & editing. JL: Data curation, Formal analysis, Methodology, Writing – original draft, Writing – review & editing. DK: Conceptualization, Data curation, Formal analysis, Funding acquisition, Investigation, Methodology, Project administration, Resources, Software, Supervision, Validation, Visualization, Writing – original draft, Writing – review & editing.
