# Supplementary material for: Microcurrent therapy as the nonpharmacological new protocol against Alzheimer’s disease
Source: Front Aging Neurosci. 2024 Jan 18;16:1344072. doi: 10.3389/fnagi.2024.1344072 (PMC10833500; doi:10.3389/fnagi.2024.1344072)
Supplement: Supplementary file 1 [file Data_Sheet_1.docx]

**Supplementary Figure Legends**

**Supplementary Fig1.**

The novel object recognition task was performed on 5xFAD mice after injection of Albumin (100μM) or Aβ(100μM). * p < 0.05

**Supplementary Fig2.**

Uncropped full image western blots and relative band intensity
