# Supplementary material for: Microcurrent therapy as the nonpharmacological new protocol against Alzheimer’s disease
Source: Front Aging Neurosci. 2024 Jan 18;16:1344072. doi: 10.3389/fnagi.2024.1344072 (PMC10833500; doi:10.3389/fnagi.2024.1344072)
Supplement: Supplementary file 2 [file Presentation_1.pptx]

## Slide 1
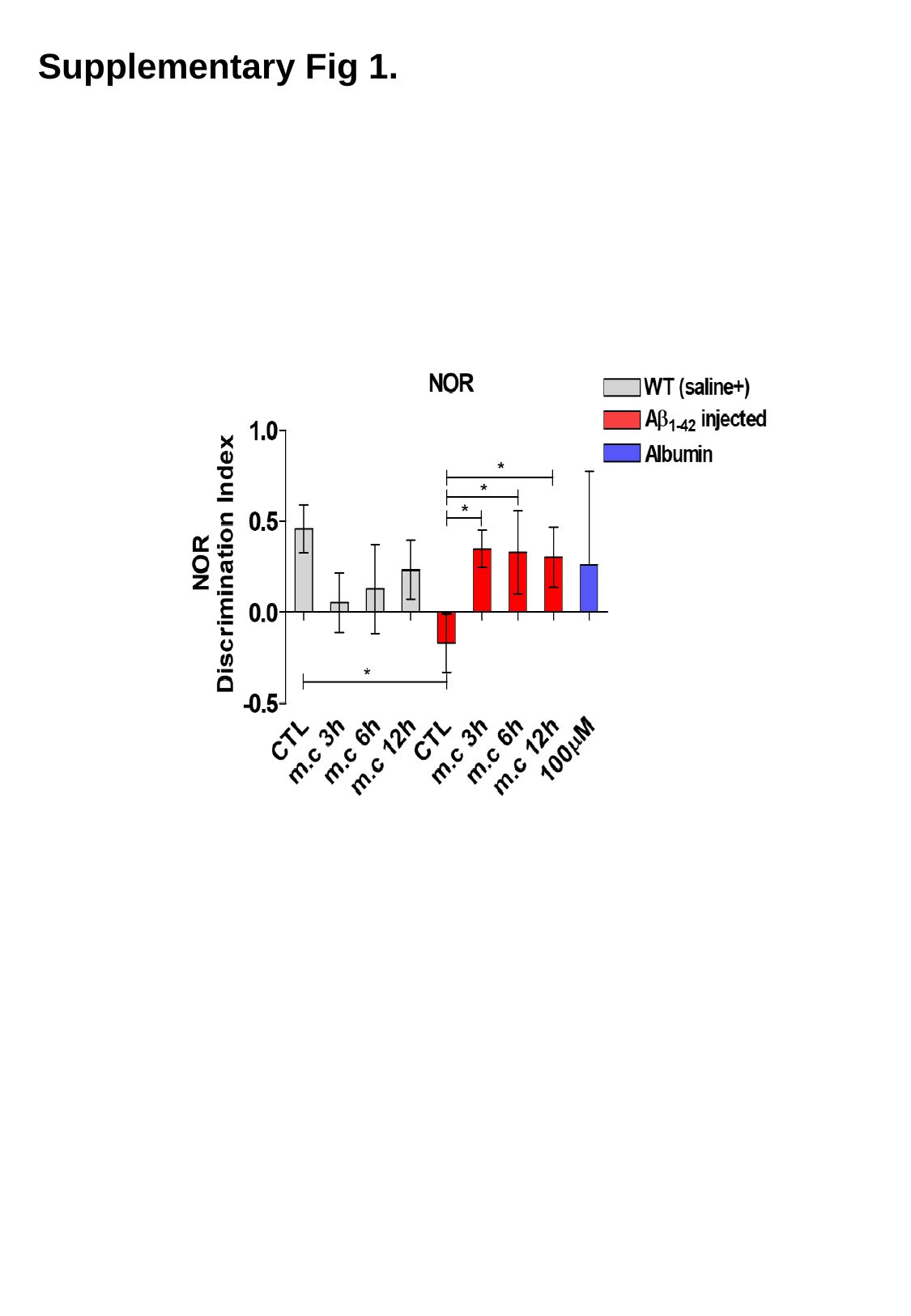

Supplementary Fig 1.

## Slide 2
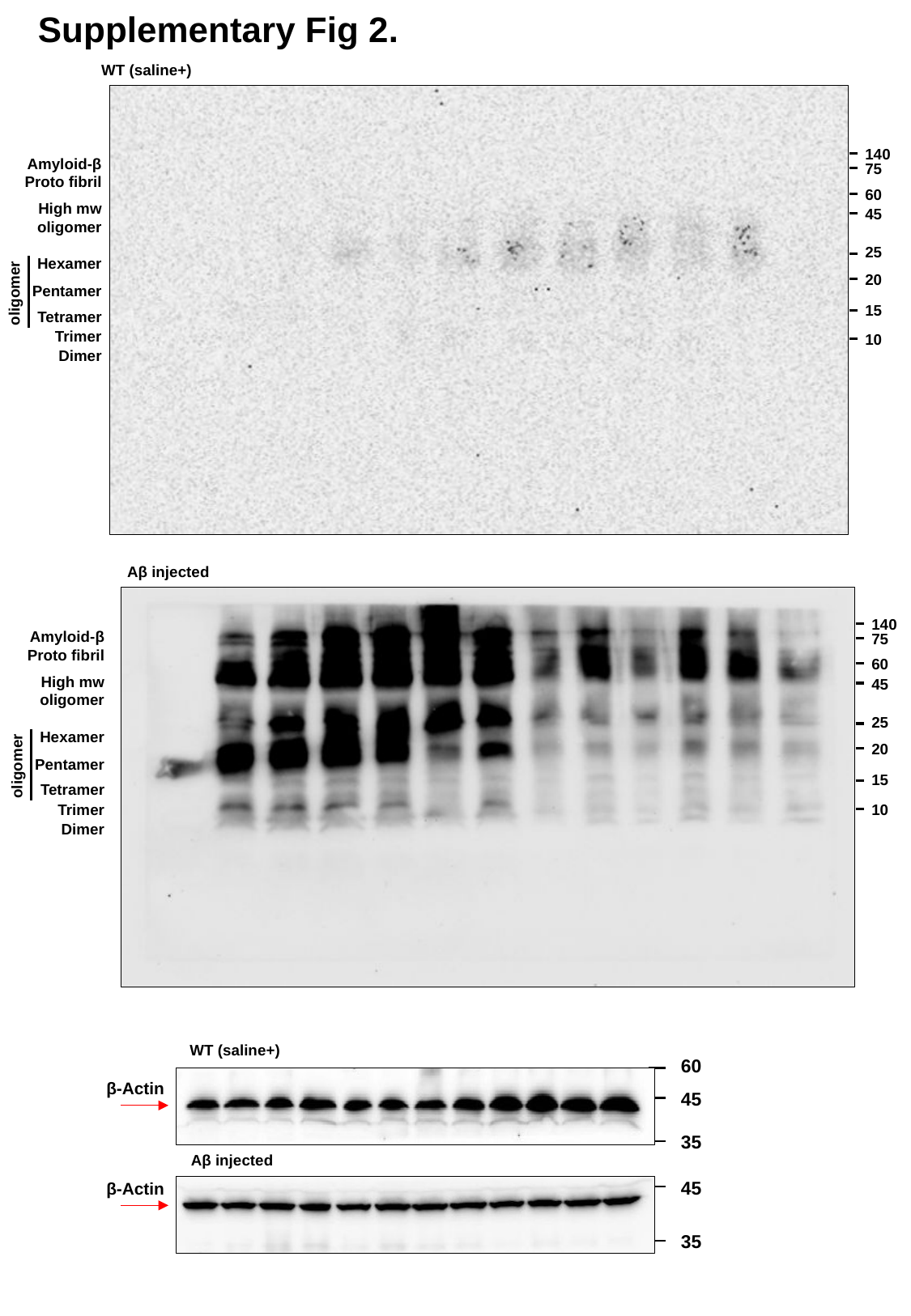

Supplementary Fig 2.
WT (saline+)
140
Amyloid-β
Proto fibril
75
60
High mw oligomer
45
25
Hexamer
20
Pentamer
oligomer
15
Tetramer
Trimer
10
Dimer
Aβ injected
140
Amyloid-β
Proto fibril
75
60
High mw oligomer
45
25
Hexamer
20
Pentamer
oligomer
15
Tetramer
Trimer
10
Dimer
WT (saline+)
60
β-Actin
45
35
Aβ injected
45
β-Actin
35

## Slide 3
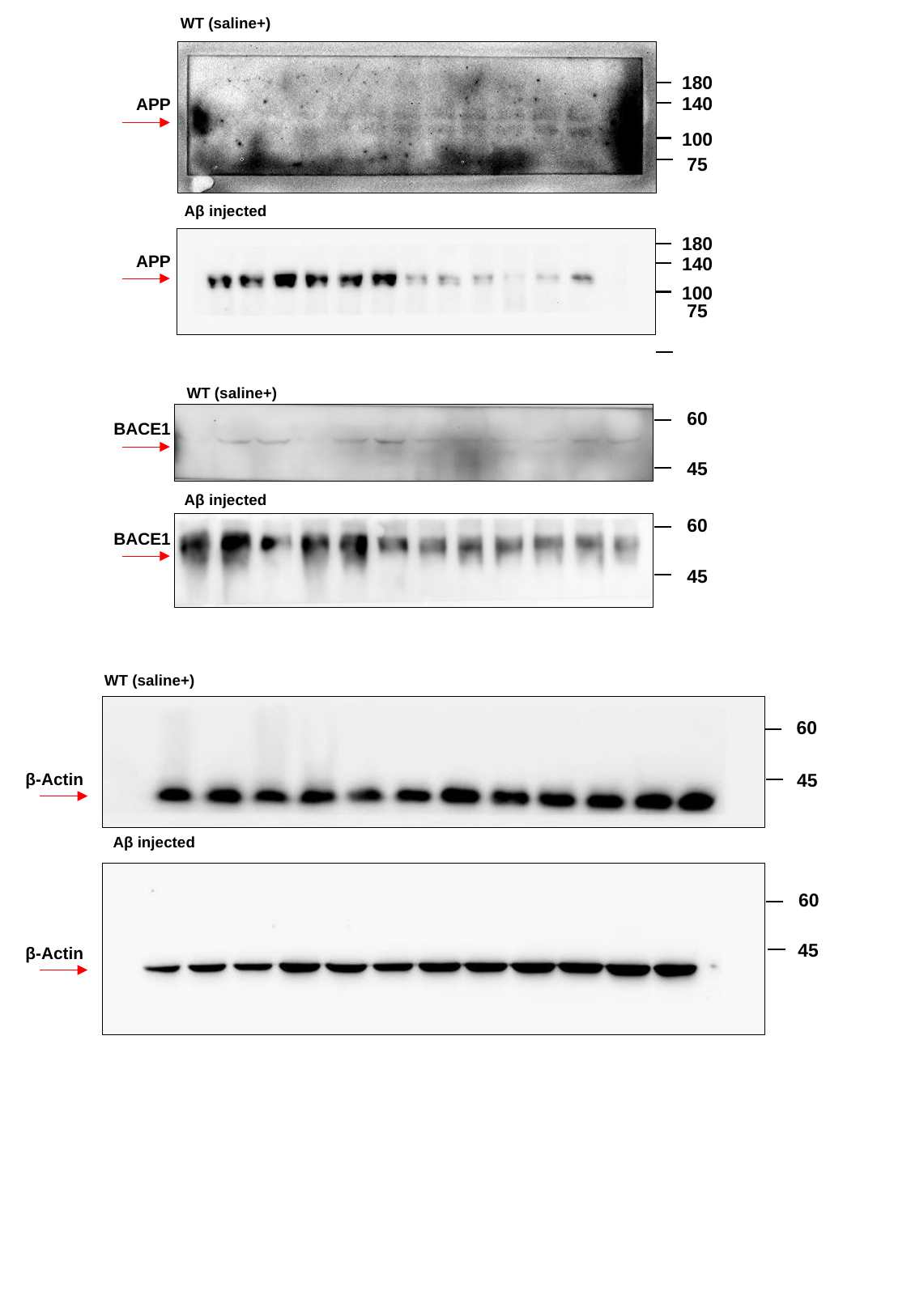

WT (saline+)
WT
180
140
APP
100
75
AD
Aβ injected
180
APP
140
100
75
WT (saline+)
60
BACE1
45
Aβ injected
60
BACE1
45
WT (saline+)
60
β-Actin
45
Aβ injected
60
45
β-Actin

## Slide 4
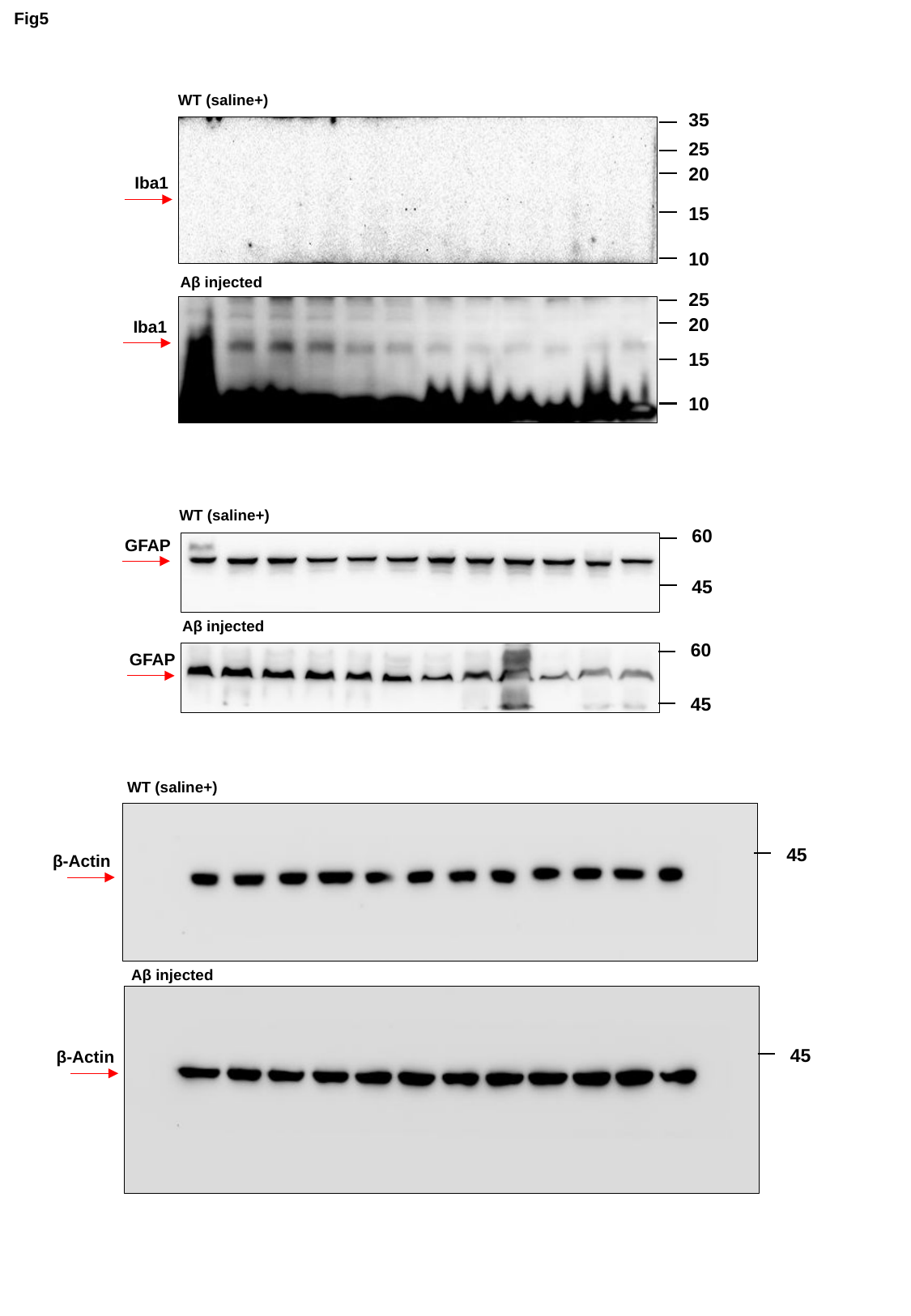

Fig5
WT (saline+)
35
25
20
Iba1
15
10
Aβ injected
25
20
Iba1
15
10
WT (saline+)
60
GFAP
45
Aβ injected
60
GFAP
45
WT (saline+)
45
β-Actin
Aβ injected
45
β-Actin

## Slide 5
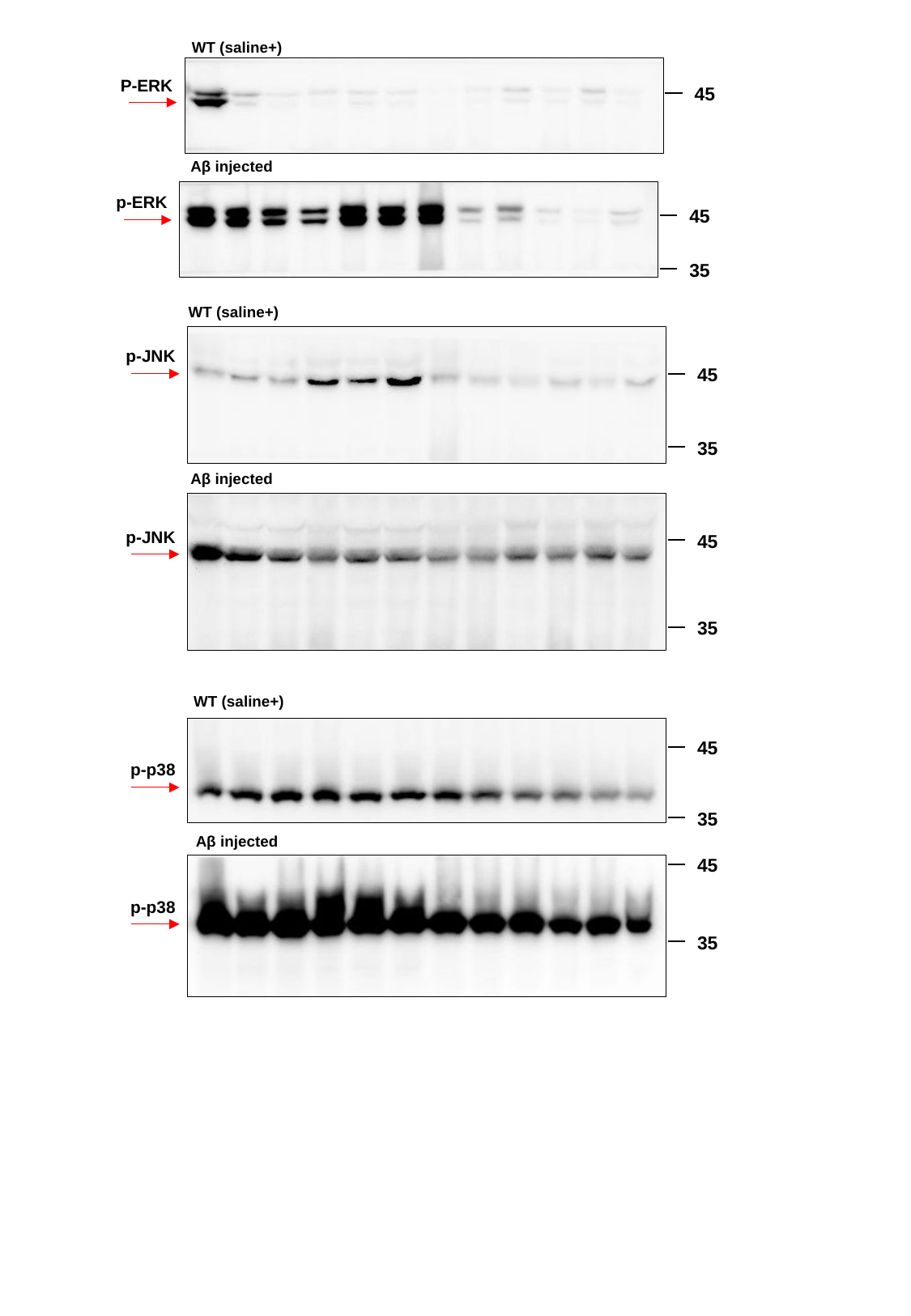

WT (saline+)
P-ERK
45
Aβ injected
p-ERK
45
35
WT (saline+)
p-JNK
45
35
Aβ injected
p-JNK
45
35
WT (saline+)
45
p-p38
35
Aβ injected
45
p-p38
35

## Slide 6
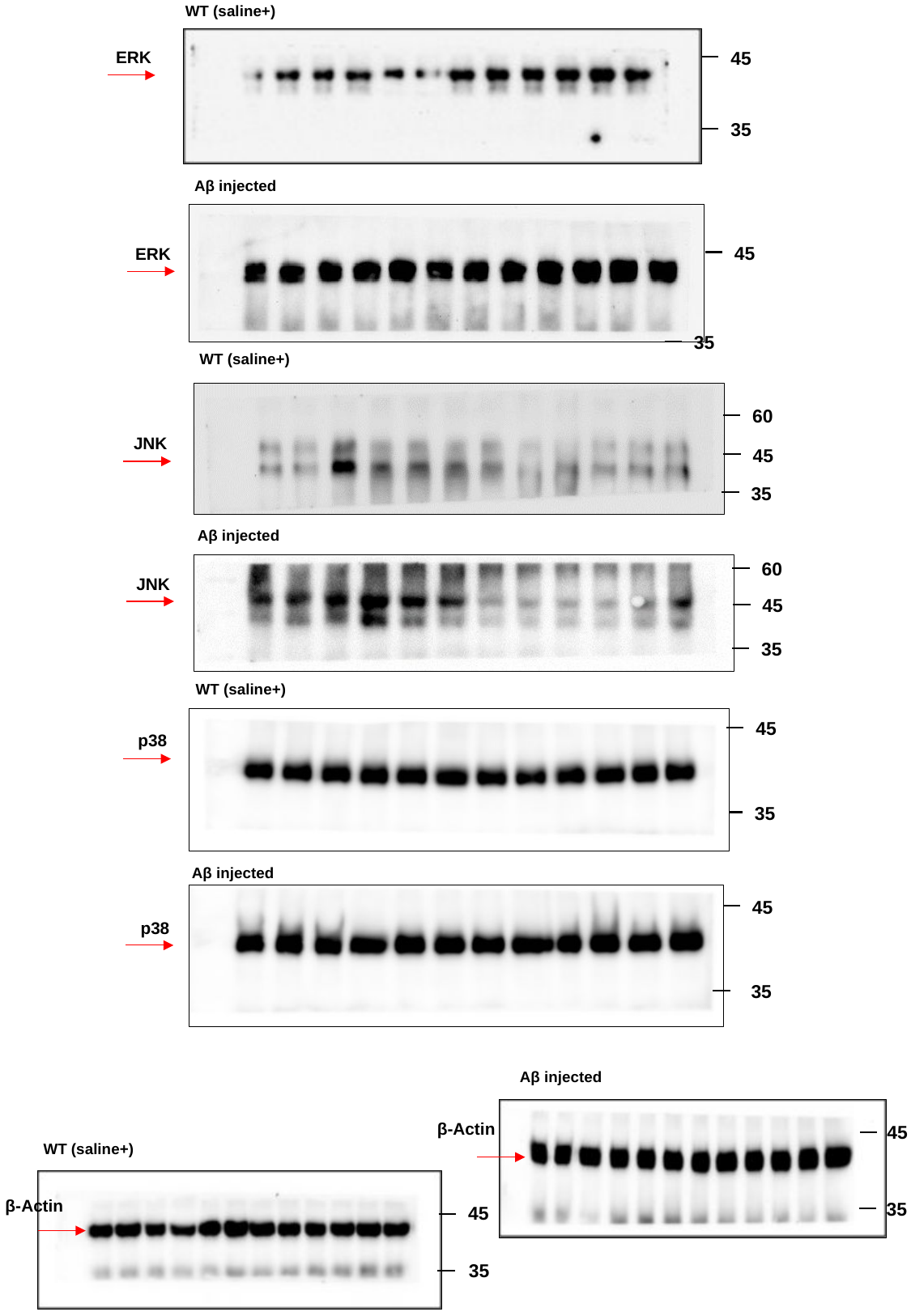

WT (saline+)
45
ERK
35
Aβ injected
45
ERK
35
WT (saline+)
60
JNK
45
35
Aβ injected
60
JNK
45
35
WT (saline+)
45
p38
35
Aβ injected
45
p38
35
Aβ injected
β-Actin
45
WT (saline+)
β-Actin
35
45
35

## Slide 7
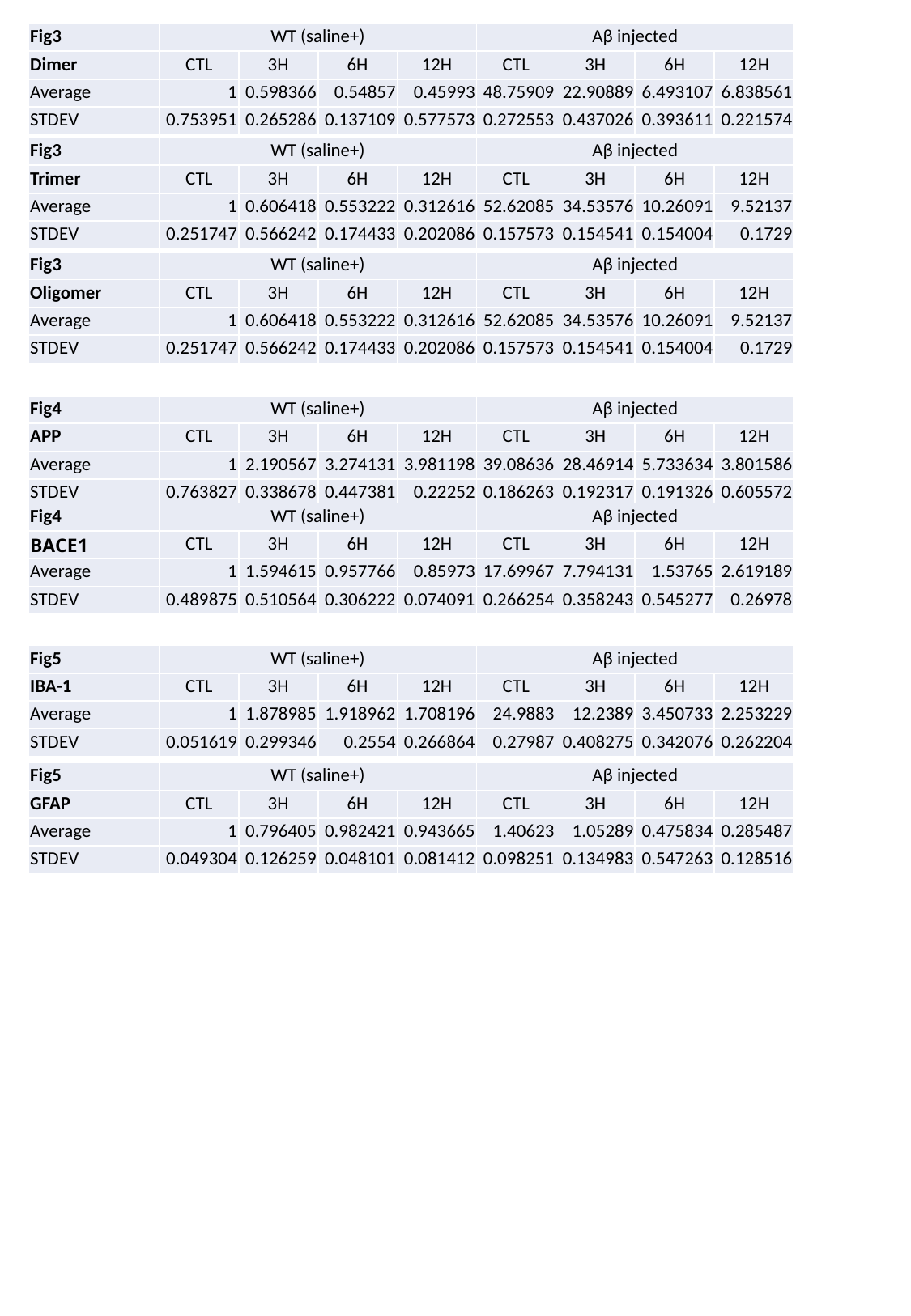

| Fig3 | WT (saline+) | | | | Aβ injected | | | |
| --- | --- | --- | --- | --- | --- | --- | --- | --- |
| Dimer | CTL | 3H | 6H | 12H | CTL | 3H | 6H | 12H |
| Average | 1 | 0.598366 | 0.54857 | 0.45993 | 48.75909 | 22.90889 | 6.493107 | 6.838561 |
| STDEV | 0.753951 | 0.265286 | 0.137109 | 0.577573 | 0.272553 | 0.437026 | 0.393611 | 0.221574 |
| Fig3 | WT (saline+) | | | | Aβ injected | | | |
| --- | --- | --- | --- | --- | --- | --- | --- | --- |
| Trimer | CTL | 3H | 6H | 12H | CTL | 3H | 6H | 12H |
| Average | 1 | 0.606418 | 0.553222 | 0.312616 | 52.62085 | 34.53576 | 10.26091 | 9.52137 |
| STDEV | 0.251747 | 0.566242 | 0.174433 | 0.202086 | 0.157573 | 0.154541 | 0.154004 | 0.1729 |
| Fig3 | WT (saline+) | | | | Aβ injected | | | |
| --- | --- | --- | --- | --- | --- | --- | --- | --- |
| Oligomer | CTL | 3H | 6H | 12H | CTL | 3H | 6H | 12H |
| Average | 1 | 0.606418 | 0.553222 | 0.312616 | 52.62085 | 34.53576 | 10.26091 | 9.52137 |
| STDEV | 0.251747 | 0.566242 | 0.174433 | 0.202086 | 0.157573 | 0.154541 | 0.154004 | 0.1729 |
| Fig4 | WT (saline+) | | | | Aβ injected | | | |
| --- | --- | --- | --- | --- | --- | --- | --- | --- |
| APP | CTL | 3H | 6H | 12H | CTL | 3H | 6H | 12H |
| Average | 1 | 2.190567 | 3.274131 | 3.981198 | 39.08636 | 28.46914 | 5.733634 | 3.801586 |
| STDEV | 0.763827 | 0.338678 | 0.447381 | 0.22252 | 0.186263 | 0.192317 | 0.191326 | 0.605572 |
| Fig4 | WT (saline+) | | | | Aβ injected | | | |
| --- | --- | --- | --- | --- | --- | --- | --- | --- |
| BACE1 | CTL | 3H | 6H | 12H | CTL | 3H | 6H | 12H |
| Average | 1 | 1.594615 | 0.957766 | 0.85973 | 17.69967 | 7.794131 | 1.53765 | 2.619189 |
| STDEV | 0.489875 | 0.510564 | 0.306222 | 0.074091 | 0.266254 | 0.358243 | 0.545277 | 0.26978 |
| Fig5 | WT (saline+) | | | | Aβ injected | | | |
| --- | --- | --- | --- | --- | --- | --- | --- | --- |
| IBA-1 | CTL | 3H | 6H | 12H | CTL | 3H | 6H | 12H |
| Average | 1 | 1.878985 | 1.918962 | 1.708196 | 24.9883 | 12.2389 | 3.450733 | 2.253229 |
| STDEV | 0.051619 | 0.299346 | 0.2554 | 0.266864 | 0.27987 | 0.408275 | 0.342076 | 0.262204 |
| Fig5 | WT (saline+) | | | | Aβ injected | | | |
| --- | --- | --- | --- | --- | --- | --- | --- | --- |
| GFAP | CTL | 3H | 6H | 12H | CTL | 3H | 6H | 12H |
| Average | 1 | 0.796405 | 0.982421 | 0.943665 | 1.40623 | 1.05289 | 0.475834 | 0.285487 |
| STDEV | 0.049304 | 0.126259 | 0.048101 | 0.081412 | 0.098251 | 0.134983 | 0.547263 | 0.128516 |

## Slide 8
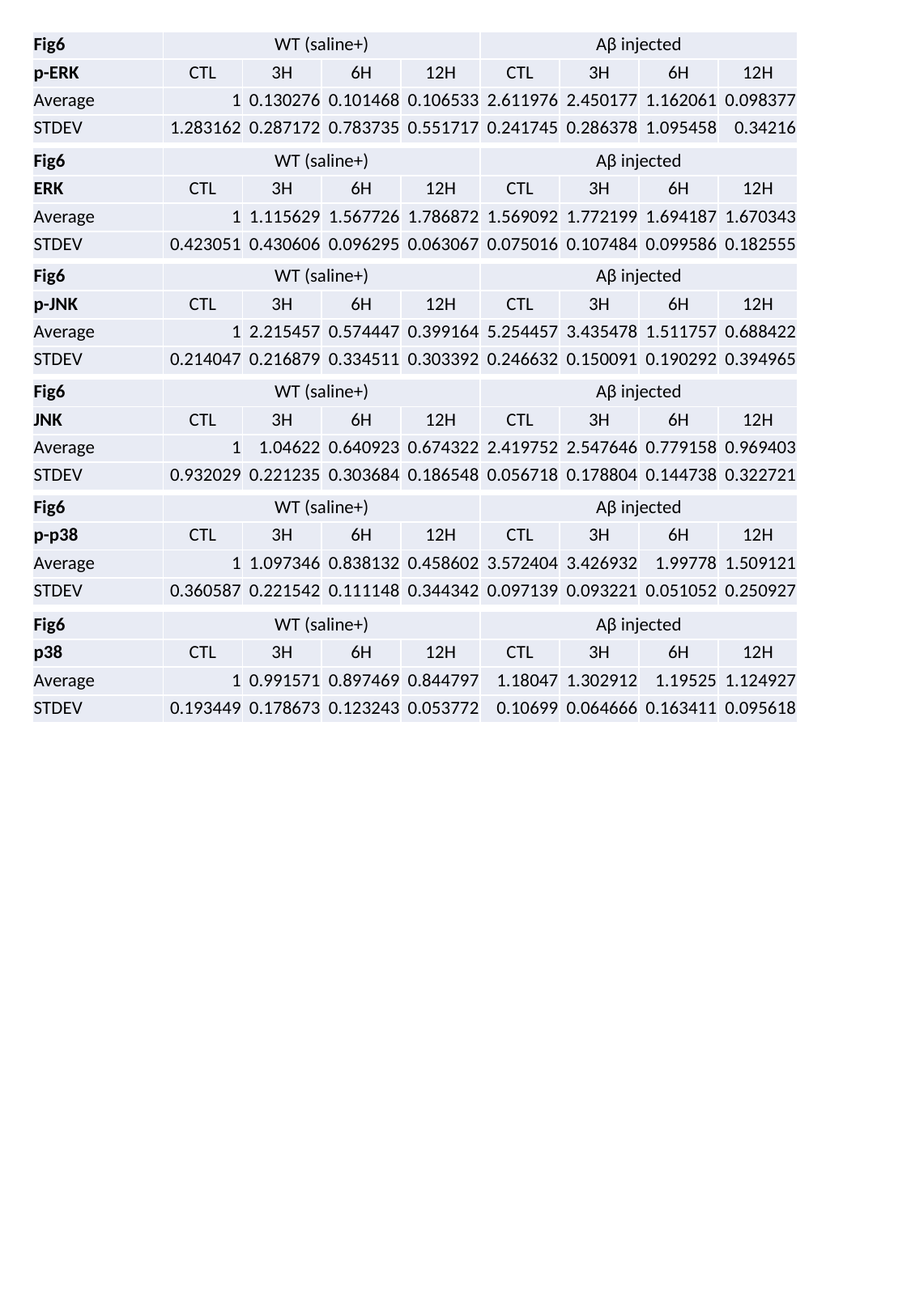

| Fig6 | WT (saline+) | | | | Aβ injected | | | |
| --- | --- | --- | --- | --- | --- | --- | --- | --- |
| p-ERK | CTL | 3H | 6H | 12H | CTL | 3H | 6H | 12H |
| Average | 1 | 0.130276 | 0.101468 | 0.106533 | 2.611976 | 2.450177 | 1.162061 | 0.098377 |
| STDEV | 1.283162 | 0.287172 | 0.783735 | 0.551717 | 0.241745 | 0.286378 | 1.095458 | 0.34216 |
| Fig6 | WT (saline+) | | | | Aβ injected | | | |
| --- | --- | --- | --- | --- | --- | --- | --- | --- |
| ERK | CTL | 3H | 6H | 12H | CTL | 3H | 6H | 12H |
| Average | 1 | 1.115629 | 1.567726 | 1.786872 | 1.569092 | 1.772199 | 1.694187 | 1.670343 |
| STDEV | 0.423051 | 0.430606 | 0.096295 | 0.063067 | 0.075016 | 0.107484 | 0.099586 | 0.182555 |
| Fig6 | WT (saline+) | | | | Aβ injected | | | |
| --- | --- | --- | --- | --- | --- | --- | --- | --- |
| p-JNK | CTL | 3H | 6H | 12H | CTL | 3H | 6H | 12H |
| Average | 1 | 2.215457 | 0.574447 | 0.399164 | 5.254457 | 3.435478 | 1.511757 | 0.688422 |
| STDEV | 0.214047 | 0.216879 | 0.334511 | 0.303392 | 0.246632 | 0.150091 | 0.190292 | 0.394965 |
| Fig6 | WT (saline+) | | | | Aβ injected | | | |
| --- | --- | --- | --- | --- | --- | --- | --- | --- |
| JNK | CTL | 3H | 6H | 12H | CTL | 3H | 6H | 12H |
| Average | 1 | 1.04622 | 0.640923 | 0.674322 | 2.419752 | 2.547646 | 0.779158 | 0.969403 |
| STDEV | 0.932029 | 0.221235 | 0.303684 | 0.186548 | 0.056718 | 0.178804 | 0.144738 | 0.322721 |
| Fig6 | WT (saline+) | | | | Aβ injected | | | |
| --- | --- | --- | --- | --- | --- | --- | --- | --- |
| p-p38 | CTL | 3H | 6H | 12H | CTL | 3H | 6H | 12H |
| Average | 1 | 1.097346 | 0.838132 | 0.458602 | 3.572404 | 3.426932 | 1.99778 | 1.509121 |
| STDEV | 0.360587 | 0.221542 | 0.111148 | 0.344342 | 0.097139 | 0.093221 | 0.051052 | 0.250927 |
| Fig6 | WT (saline+) | | | | Aβ injected | | | |
| --- | --- | --- | --- | --- | --- | --- | --- | --- |
| p38 | CTL | 3H | 6H | 12H | CTL | 3H | 6H | 12H |
| Average | 1 | 0.991571 | 0.897469 | 0.844797 | 1.18047 | 1.302912 | 1.19525 | 1.124927 |
| STDEV | 0.193449 | 0.178673 | 0.123243 | 0.053772 | 0.10699 | 0.064666 | 0.163411 | 0.095618 |
